# Estimates of the incidence, prevalence, and factors associated with common sexually transmitted infections among Lebanese women

**DOI:** 10.1371/journal.pone.0301231

**Published:** 2024-04-18

**Authors:** Hiam Chemaitelly, Ramzi R. Finan, Eddie Racoubian, Gulzhanat Aimagambetova, Wassim Y. Almawi

**Affiliations:** 1 Infectious Disease Epidemiology Group, Weill Cornell Medicine-Qatar, Cornell University, Doha, Qatar; 2 Department of Population Health Sciences, Weill Cornell Medicine, Cornell University, Ithaca, New York, United States of America; 3 Department of Obstetrics and Gynecology, Hôtel Dieu de France, CHU Université St. Joseph, Beirut, Lebanon; 4 St. March Medical and Diagnostic Center, Beirut, Lebanon; 5 Department of Surgery, School of Medicine, Nazarbayev University, Astana, Kazakhstan; 6 Department of Biological Sciences, Brock University, St. Catharines, Ontario, Canada; 7 Faculty of Sciences, El Manar University, Tunis, Tunisia; Universidade Federal do Espirito Santo, BRAZIL

## Abstract

**Background:**

We analyzed the prevalence of active infection with common curable sexually transmitted infections (STIs) including *N*. *gonorrhea*, *C*. *trachomatis*, *T*. *vaginalis*, and *T*. *pallidum*, as well as active infection with HPV, herpes simplex virus types I (HSV-1) and II (HSV-2), *M*. *hominis*, *M*. *genitalium*, *C*. *albicans*, and Ureaplasma in 351 Lebanese women.

**Methods:**

A cross-sectional study, involving 351 sexually active women, 40 years or younger, who were recruited from outpatient Obstetrics and Gynecology clinic attendees between September 2016 and November 2017.

**Results:**

The prevalence of active infection was low at 0.3% for *N*. *gonorrhea*, 0.6% for HSV-2, 2.8% for *C*. *trachomatis*, and 2.9% for any curable STIs. Prevalence of active HPV infection was high assessed at 15.7% for high-risk and 12.2% for low-risk genotypes. Furthermore, the prevalence was 2.0% for *M*. *genitalium*, 6.8% for ureaplasma, 13.7% for *Candida albicans*, and 20.5% for *M*. *hominis*. No active infections with *T*. *vaginalis*, *T*. *pallidum*, or HSV-1 were observed. Significant age differences were noted in the prevalence of high-risk and low-risk HPV genotypes, but no such differences were noted in the prevalence of other infections. No appreciable variations were identified in the prevalence of key STIs based on smoking, marital status, or the number of sexual partners.

**Conclusions:**

The study documented active infection with substantial prevalence for multiple STIs among women attending outpatient gynecology and obstetrics clinics in Lebanon. These findings underscore the importance of strengthening STI surveillance, linkage to care, and prevention interventions in reducing STI incidence among women.

## Introduction

Sexually transmitted infections (STIs) remain a global pervasive public health challenge, with women across wide age groups, and socioeconomic and geographical backgrounds, bearing a significant portion of this burden [1, 2]. STIs encompass an array of bacterial, viral, and parasitic infections that are transmitted through sexual contact, and range from the common chlamydia and gonorrhea to the more serious and likely life-threatening infections such as HIV/AIDS [2, 3]. Several factors are associated with the persistence of STIs, which include changes in sexual behavior, lack of sufficient awareness, evolving pathogens, inadequate access to healthcare, and stigma [4, 5]. STIs differ in their scope, characteristics, prognosis, and consequences, which can vary from localized inflammatory disorder to cancer, and even death [4, 6], thus necessitating comprehensive STI investigation and testing in women at a young age, and periodically thereafter.

Infection with STIs can result in a range of associated complications if left untreated [7, 8]. These include cervical cancer, infertility, chronic pain, and heightened neonatal morbidity and mortality [2, 9]. Accurate and timely detection of active STIs is crucial for the affected individual and public health management [9, 10]. The advent of DNA/RNA-based PCR has allowed for highly sensitive and specific detection of STI pathogens in different sample types, including blood, urine, and vaginal discharges [9, 11]. Despite the availability of ultra-sensitive diagnostic tests for STI causative agents, the prevalence of STIs remains a public health concern, both in developed, developing, and under-developed countries [2, 6].

Lebanon is situated in the East Mediterranean, and its present-day population comprise a mix of Christian (Catholic/Maronite, Greek Orthodox, Copts) and Moslem (Sunni, Shiite, Druze) communities [12, 13]. Several socio-cultural factors including limited sex education, gender inequality, social norms and beliefs, and high-risk sexual behaviors, along with healthcare issues and challenges, comprising limited access to and cost of healthcare, deteriorating healthcare system, and lack of comprehensive STI services contribute to the increase in the STI prevalence [14]. This is compounded by the recent refugee crisis [15, 16], emerging STI strains, and limited data collection and surveillance, thus highlighting the need for a comprehensive approach aimed at providing access to affordable healthcare services and strengthening STI surveillance [14, 16].

This study investigated the prevalence of active infection with *N*. *gonorrhea*, *C*. *trachomatis*, *T*. *vaginalis*, *T*. *pallidum*, Human papillomavirus (HPV), herpes simplex virus types I (HSV-1) and II (HSV-2), *M*. *hominis*, *M*. *genitalium*, *C*. *albicans*, and ureaplasma in 351 Lebanese women attending outpatient obstetrics and gynecology (OB/GYN) clinics, of whom 291 were 40 years or younger. The study further explored the variations in prevalence by age, smoking status, marital status, and number of sexual partners.

## Subjects and methods

### Study participants

The study participants comprised 351 women who were attending the outpatient OB/GYN clinics at Hôtel-Dieu de France Hospital and St. Marc Medical Center in the Greater Beirut area, between September 1, 2016, and November 30, 2017. All women presented to the clinic for routine gynecological check-up and were asymptomatic, and none reported pregnant at the time she was invited to participate in the study. A written informed consent was obtained from consenting participants.

### Data collection

Patients’ socio-demographic and risk behavior data were retrieved through a review of medical charts, and a unified questionnaire that details the demographic characteristics, sexual behaviors, history of STIs, and other relevant factors. The confidentiality of the participants was strictly maintained throughout the study, which included procedures for data storage handling and access, and anonymization.

### Specimens’ collection

Endocervical specimens and first-catch urine specimens were then collected and processed within 4 hours of collection. Swabs were collected by a gynecologist using speculum-assisted Spatula of Ayre after removing the mucus and were placed in a balanced saline solution. Total genomic DNA was extracted by the mini-spin column method (Qiagen, Hilden, Germany). DNA from urine specimens was extracted by the Roche Amplicor STD specimen preparation kit (Roche Diagnostics, Mannheim, Germany), according to the manufacturer’s specifications.

### HPV DNA amplification

HPV testing on endocervical DNA samples was done by nested PCR using MY09 and MY11 external primers and GP05+/GP06+ internal primers, as shown elsewhere [7]. HPV genotypes were detected by HPV Quant-21® (DNA Technology, Moscow, Russian Federation), and were grouped into low-risk (LR) (HPV6, 11, 42, 43, 44, and 70), and high-risk (HR) (HPV16, 18, 26, 31, 33, 35, 39, 45, 51, 52, 53, 56, 58, 59, 66, 68, 73, and 82). Detection of other STIs was also done using a specific DNA Technology Detection kit (DNA Technology).

### Statistical analysis

Participant characteristics were summarized using both frequency distributions and measures of central tendency. In cases where specific variables had missing values (constituting ≤2.2% of the dataset), these values were imputed using the median of observed outcomes for individuals with complete data. Descriptive statistics were performed on all testing samples, and the prevalence of active infections, along with the corresponding 95% confidence intervals (CI), were estimated. Stratified analyses were conducted to investigate the distribution of active infections across various factors, including age, smoking status, marital status, and number of sexual partnerships. Chi-square tests were performed to assess the association of each covariate with active infection. A p-value below 0.05 indicated a strong association with active infection. Due to the limited number of PCR-positive samples for *N*. *gonorrhea*, *C*. *trachomatis*, *T*. *vaginalis*, and *T*. *pallidum* (syphilis), these infections were combined to define the presence of any curable STI. To gain deeper insights into the determinants of active infection with key STIs, exploratory univariate and multivariate regression analyses were performed. However, these analyses had insufficient statistical power to detect statistical significance. All statistical analyses were conducted using Stata/SE version 17.0 (Stata Corporation, College Station, TX, USA).

### Ethical statement

This study was performed in line with the principles of the Declaration of Helsinki. Approval was granted by the Research and Ethics Committee of St. Marc Medical Center (Number, SMMC-2019-0047), granted on October 17, 2019. This study was reported according to the Strengthening the Reporting of Observational Studies in Epidemiology (STROBE) guidelines (S1 Table).

## Results

### Study population

The study included 351 women who underwent testing for an active STI infection. Table 1 shows the characteristics of the study participants. The median age was 33.0 years, with an interquartile range (IQR) of 29.0–37.0 years. Approximately 70% were married, and the rest were single, divorced, or separated. A third of women identified as smokers. About two-thirds reported up to one sexual partner, whereas 15.9% had two sexual partners, and 25.4% had three or more sexual partners.

**Table 1:** Characteristics of study participants.

Characteristics	Categories	N (%)
Total sample size		351 (100.0)
Age (years)	Median (IQR) ^1^	33.0 (29.0–37.0)
Age—years	20–29 years	100 (28.5)
20–29 years	30–39 years ^2^	191 (54.4)
30–39 years*	40–49 years	49 (14.0)
40–49 years	50+ years	11 (3.1)
Smoking		110 (31.2)
Marital	Married	240 (68.4)
	Single/Divorced/Separated ^3^	111 (31.6)
Sexually active		334 (95.2)
Numbers of partners	0–1 partner ^4^	206 (58.7)
	2 partners	56 (15.9)
	3+ partners	89 (25.4)
HSV-1		0 (0.0)
HSV-2		2 (0.6)
*Neisseria gonorrhoeae*		1 (0.3)
*Chlamydia trachomatis*		10 (2.8)
*Trichomonas vaginalis*		0 (0.0)
*Treponema pallidum*		0 (0.0)
*Mycoplasma hominis*		72 (20.5)
*Mycoplasma genitalium*		7 (2.0)
HPV-High risk		55 (15.7)
HPV-Low risk		43 (12.2)
*Candida albicans*		48 (13.7)
Ureaplasma		24 (6.8)

**IQR**, interquartile range.

1. 8 observations were imputed at the median age of 33 years.

2. Includes 6 missing observations.

3. Includes 7 missing observations.

### Active infection prevalence

**Fig 1:**
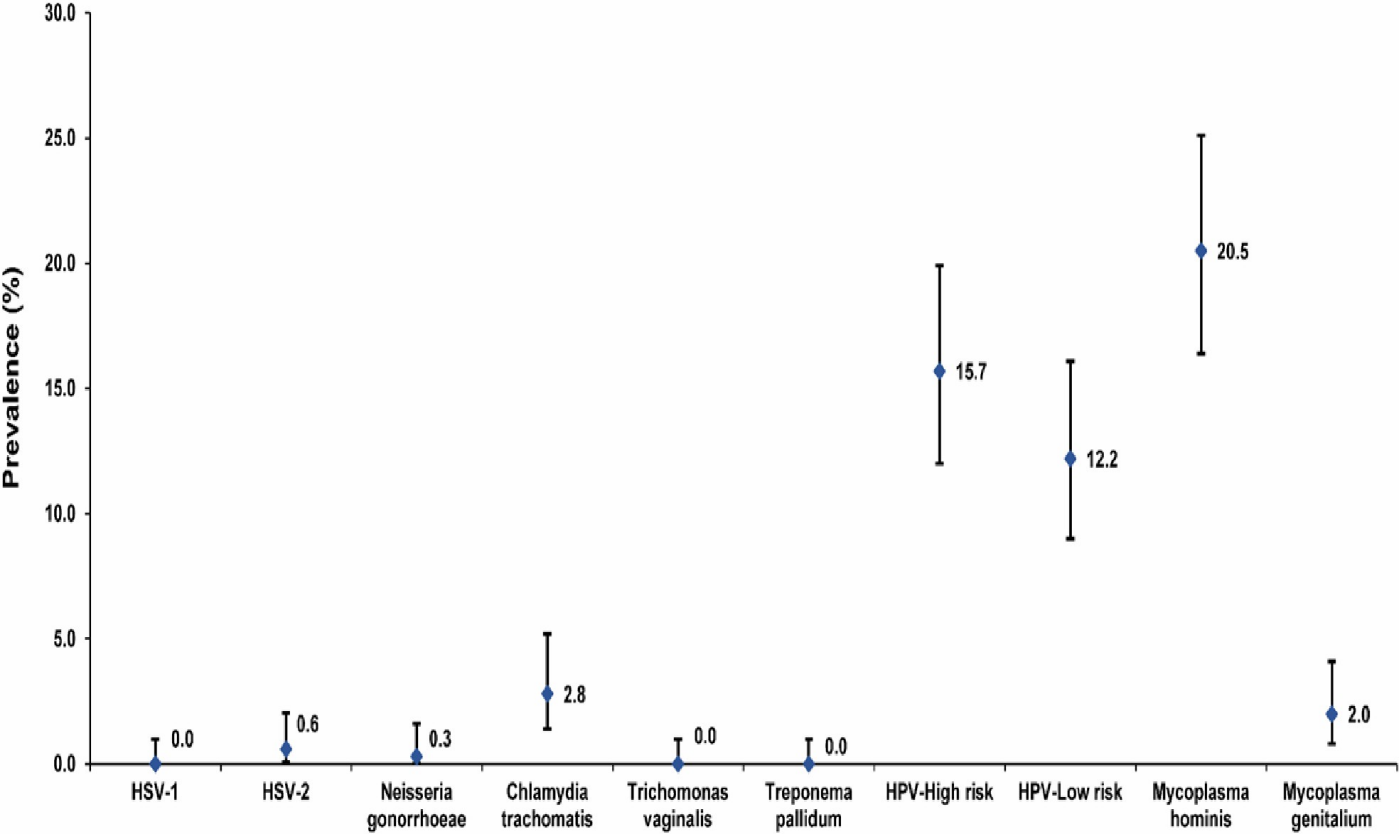
Prevalence of active infection with sexually transmitted infections among women gynecology clinic attendees in Beirut, Lebanon.

The prevalence of active infections among study participants (as identified by PCR) was nil (95% CI: 0.0–1.0%) for HSV-1 and low at 0.6% (95% CI: 0.1–2.0%) for HSV-2, indicating minimal instances of active viral shedding (Fig 1). While the prevalence for any curable STI was 2.9% (95% CI: 1.4–5.2%), the prevalence for curable bacterial STIs was 0.3% (95% CI: 0.0–1.6%) for *N*. *gonorrhea*, 2.8% (95% CI: 1.4–5.2%) for *C*. *trachomatis*, and nil (95% CI: 0.0–1.0%) for each of *T*. *vaginalis* and *T*. *pallidum*. Active HPV infection was common with a prevalence of 15.7% (95% CI: 12.0–19.9%) for HR genotypes and 12.2% (95% CI: 9.0–16.1%) for LR genotypes. Prevalence was 2.0% (95% CI: 0.8–4.1%) for *M*. *genitalium* and 20.5% (95% CI: 16.4–25.1%) for *M*. *hominis*, 6. Compared to the rates of 8% (95% CI: 4.4–10.0%) seen for Ureaplasma and 13.7% (95% CI: 10.3–17.7%) for *Candida albicans* (Fig 1).

### Patterns of active infection

**Fig 2:**
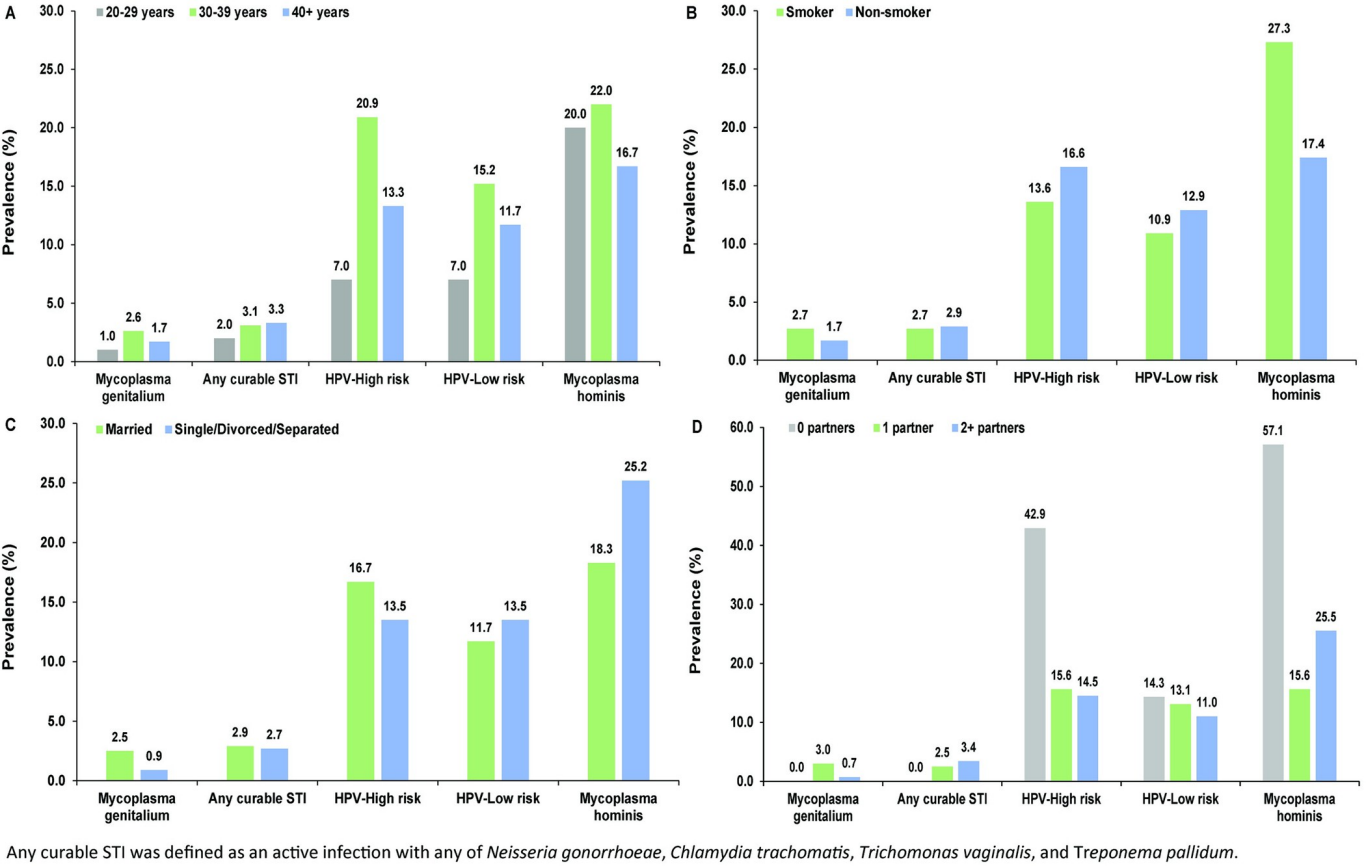
Prevalence of active infection with sexually transmitted infections A) by age group, B) smoking status, C) marital status, and D) number of sexual partners among women gynecology clinic attendees in Beirut, Lebanon. Any curable STI was defined as an active infection with any of *Neisseria gonorrhoeae*, *Chlamydia trachomatis*, *Trichomonas vaginalis*, and *Treponema pallidum*.

Fig 2 illustrates the distribution of active infection prevalence by age, smoking status, marital status, and number of sexual partners, and detailed statistical associations are provided in S2–S4 Tables. There were negligible variations in prevalence across age groups for *M*. *genitalium* and for any curable STI, with prevalence ranging from 1.0–2.6% (p-value: 0.632) and 2.0–3.3% (p-value: 0.831), respectively (Fig 2A). A similar pattern was observed for *M*. *hominis* in the face of the higher prevalence rates, which ranged from 16.7–22.0% (p = 0.665). Meanwhile, significant age differences were noted in the prevalence of HR HPV, which was lowest in women aged 20–29 years (7.0%) and highest in women aged 30–39 years (20.9%) (p = 0.007). Prevalence of LR HPV also varied in women aged 20–29 years (7.0%) compared to 30–39-year-old women (15.2%) (p = 0.128).

No appreciable differences were observed in the prevalence of key STIs by smoking status (Fig 2B), except for *M*. *hominis* where the prevalence was 27.3% among smokers compared to 17.4% among non-smokers (p-value: 0.034). Negligible differences in prevalence were also noted by marital status across all STIs (Fig 2C), despite the prevalence of *M*. *hominis* being much higher in single, divorced, and separated women (25.2%) compared to married women (18.3%). Minimal variation in the prevalence of key STIs was also found for women reporting none, one, and two or more sexual partners (Fig 2D). The prevalence among women reporting 0 partners reflects the underreporting of partnerships for social desirability. These results confirm the findings of our main analysis of substantial STI prevalence that goes undetected among women regardless of the reported number of partners. Of note, the prevalence of *M*. *hominis* was substantially higher in the latter (25.5%) compared to the former group (17.0%; p-value: 0.051).

## Discussion

Our assessment of active STI infections in a group of women attending OB/GYN clinics revealed prevalence levels for common STIs that align with global estimates. These prevalence levels are however higher than expected, given the generally conservative sexual norms in the Middle East and North Africa (MENA) region. Specifically, the prevalence of *N*. *gonorrhoeae* and *C*. *trachomatis* were 0.3% and 2.8%, respectively, within the CIs of the global prevalence for these infections estimated by the WHO at 0.8% and 3.2%, respectively [17]. The prevalence of HR (15.7%) and LR (12.2%) HPV genotypes were also considerable, comparable to levels observed in Asia, but lower than levels observed in developed countries such as Europe and North America where routine screening for HPV is implemented [18].

The higher-than-expected prevalence identified among women may not necessarily imply increased engagement in sexual risk behaviors [14, 16]. Instead, these findings underscore the lack of sexual health services and the limited capacity for STI prevention and treatment in Lebanon, and the broader MENA region [15, 19]. Socio-cultural sensitivities, constrained resources, and competing national health priorities have contributed to STIs being neglected in research and surveillance, as well as on national public health agendas [19, 20]. Consequently, infections often go undetected, persisting for prolonged periods, and heightening the potential for infection circulation within the population [16, 19]. This situation significantly elevates the risk of future health complications among women [19, 21].

The minimal variations in active infection levels by smoking status, marital status, and the number of lifetime sexual partners affirm the notion that insufficient access to STI services is the primary factor contributing to the higher-than-expected prevalence levels [14]. In particular, the prevalence among women reporting 0 partners reflects the underreporting of number of sexual partnerships for social considerations (stigma and shame) and taboos surrounding sexuality. These results strongly suggest substantially higher STI prevalence that goes undetected among women regardless of the self-reported number of partners. Our findings showed variability in HR and LR HPV across age groups, with the most elevated prevalence found among women aged 30–39 years [22]. This pattern could signify an increased likelihood of virus exposure over time. However, it may also be influenced by a survival bias, where women in older age groups, having a greater chance of progressing to cervical cancer, are less likely to have survived the complications associated with HPV. These findings are consistent with existing literature indicating the highest prevalence of HPV in this age group, as well as the increased incidence of cervical cancer among older women [23, 24].

There is a pressing need to integrate HPV screening into routine assessments for women of reproductive age in Lebanon and other Middle East and North Africa region (MENA) countries or, at the very least, to enhance awareness about the significance of HPV screening among women [25], akin to the campaigns established for breast cancer awareness [14, 25, 26]. Several cultural, economic, and access to healthcare factors contribute to the rising, but not fully documented, prevalence of STIs [6, 19, 21]. These include cultural and religious norms that lead to reluctance to seek testing and treatment for STIs, and conservative attitudes in those communities, which limit the ability of women to negotiate safer sex practices [20, 25, 30]. Socioeconomic factors, coupled with the cost of and limited access to healthcare services, and an emerging influx of refugees have widened the disparities in access to healthcare and efficient STI services [15, 16, 19, 20].

Co-infections of HPV-infected women with other STIs, namely Chlamydia, Mycobacteria, and HIV, were linked to HPV persistence, and increased cervical neoplasia risk [4, 27]. Insofar as they were reported for different ethnic groups and pathophysiological conditions [3, 28], this suggested a geographical pattern of co-infection [10, 29], likely linked with socioeconomic and education status and inadequate access to healthcare facilities [5, 7, 30]. The majority of women with co-infections were positive for HPV (8.6%) as part of double infection with mostly *M*. *hominis*, which is attributed to the high prevalence of these two STIs globally. This was in line with findings on Nigerian women, in which persistent *M*. *hominis* infection was linked with HPV positivity, without establishing a clear cause-and-effect link [31]. Other coinfections included mycoplasma with chlamydia and candida, which were not as pronounced as HPV coinfections, in agreement with a recent German study [30]. However, co-infection prevalence was independently associated with smoking and having multiple sexual partners [4, 7].

## Strengths and limitations

This study has limitations. The term “co-infection” was based on the detection of nucleic acid material without indication of the actual disease status. Women were recruited from outpatient clinics in a hospital located in the capital city, and therefore our findings may not be generalizable to the broader female population in the country [32]. The number of identified positive cases was relatively small hindering the conduct of meaningful regression analyses. The limited sample size restricted analysis of complete stratification based on the number of sexual partners. Despite these shortcomings, this study provided evidence of the current prevalence of a broad spectrum of infections among women in the general population, shedding light on a neglected disease burden.

## Conclusions

In conclusion, our study revealed the higher-than-expected prevalence of STIs among women attending OB/GYN clinics in the MENA region, emphasizing the significance of reinforcing STI surveillance to develop a better understanding of the burden of these infections nationwide and monitor infection trends over time [17]. Our findings also underscore the imperative for targeted prevention interventions, including HPV immunization [26], enhanced access to sexual health services, integration of STIs into routine screening protocols, and improved linkage to care [14, 25]. Implementing these strategies is essential to reducing STI incidence and mitigating their adverse impact on women’s health, social well-being, and economic welfare.

## Supporting information

S1 Checklist Human participants research checklist. (DOCX)

S1 Data (XLSX)

S1 Table STROBE guidelines. (DOCX)

S2 Table Associations with active infection with any of *Neisseria gonorrhoeae*, *Chlamydia trachomatis*, *Trichomonas vaginalis*, and *Treponema pallidum*. (DOCX)

S3 Table Associations with active infection with human papillomavirus (HPV). (DOCX)

S4 Table Associations with active infection with *Mycoplasma hominis* and *Mycoplasma genitalium*. (DOCX)

## References

[1] Mohammed H, Hughes G, Fenton KA. Surveillance systems for sexually transmitted infections: a global review. Curr Opin Infect Dis. 2016;29: 64–69. doi: 10.1097/QCO.0000000000000235 26658655

[2] Unemo M, Bradshaw CS, Hocking JS, de Vries HJC, Francis SC, Mabey D, et al. Sexually transmitted infections: challenges ahead. *Lancet Infect Dis*. 2017;17: e235–e279. doi: 10.1016/S1473-3099(17)30310-9 28701272

[3] Menezes LJ, Pokharel U, Sudenga SL, Botha MH, Zeier M, Abrahamsen ME, et al. Patterns of prevalent HPV and STI co-infections and associated factors among HIV-negative young Western Cape, South African women: the EVRI trial. Sex Transm Infect. 2018;94: 55–61. doi: 10.1136/sextrans-2016-053046 28490581 PMC6561095

[4] Kops NL, Bessel M, Horvath JDC, Domingues C, de Souza FMA, Benzaken AS, et al. Factors associated with HPV and other self-reported STI coinfections among sexually active Brazilian young adults: cross-sectional nationwide study. BMJ Open. 2019;9: e027438. doi: 10.1136/bmjopen-2018-027438 31230011 PMC6596954

[5] Menezes ME, Silver EJ, Goldstein DY, Collins-Ogle MD, Fox AS, Coupey SM. Prevalence and factors associated with Mycoplasma genitalium infection in at-risk female adolescents in Bronx County, New York. Sex Transm Dis. 2023;50: 635–641. doi: 10.1097/OLQ.0000000000001840 37255234

[6] Newman L, Rowley J, Vander Hoorn S, Wijesooriya NS, Unemo M, Low N, et al. Global estimates of the prevalence and incidence of four curable sexually transmitted infections in 2012 based on systematic review and global reporting. PLoS One. 2015;10: e0143304. doi: 10.1371/journal.pone.0143304 26646541 PMC4672879

[7] Finan RR, Musharrafieh U, Almawi WY. Detection of Chlamydia trachomatis and herpes simplex virus type 1 or 2 in cervical samples in human papillomavirus (HPV)-positive and HPV-negative women. Clin Microbiol Infect. 2006;12:927–930. doi: 10.1111/j.1469-0691.2006.01479.x 16882302

[8] Gökengin D, Noori T, Alemany A, Bienkowski C, Liegon G, İnkaya AÇ, et al. Prevention strategies for sexually transmitted infections, HIV, and viral hepatitis in Europe. *Lancet Reg Health Eur*. 2023 Oct 26;34:100738. doi: 10.1016/j.lanepe.2023.100738 37927439 PMC10625023

[9] Adamson PC, Loeffelholz MJ, Klausner JD. Point-of-care testing for sexually transmitted infections: A review of recent developments. *Arch Pathol Lab Med*. 2020 Nov 1;144(11): 1344–1351. doi: 10.5858/arpa.2020-0118-RA 32810868 PMC7606737

[10] Elmi AA, Bansal D, Acharya A, Skariah S, Dargham SR, Abu-Raddad LJ, et al. Human papillomavirus (HPV) infection: molecular epidemiology, genotyping, seroprevalence and associated risk factors among Arab women in Qatar. *PLoS One*. 2017 Jan 3;12(1):e0169197. doi: 10.1371/journal.pone.0169197 28046025 PMC5207789

[11] Finan RR, Irani-Hakime N, Tamim H, Almawi WY. Molecular diagnosis of human papillomavirus: comparison between cervical and vaginal sampling. *Infect Dis Obstet Gynecol*. 2001;9(2):119–22. doi: 10.1155/S1064744901000217 11495553 PMC1784642

[12] Almawi WY, Busson M, Tamim H, Al-Harbi EM, Finan RR, Wakim-Ghorayeb SF, et al. HLA class II profile and distribution of HLA-DRB1 and HLA-DQB1 alleles and haplotypes among Lebanese and Bahraini Arabs. Clin Diagn Lab Immunol. 2004 Jul;11(4):770–4. doi: 10.1128/CDLI.11.4.770-774.2004 15242955 PMC440602

[13] Hajjej A, Almawi WY, Arnaiz-Villena A, Hattab L, Hmida S. The genetic heterogeneity of Arab populations as inferred from HLA genes. PLoS One. 2018 Mar 9;13(3):e0192269. doi: 10.1371/journal.pone.0192269 29522542 PMC5844529

[14] Maatouk I, Assi M, Jaspal R. Predicting sexual risk and sexual health screening in a sample of university students in Lebanon: a cross-sectional study. J Am Coll Health. 2023 Feb-Mar;71(2):593–599. doi: 10.1080/07448481.2021.1899188 33830876

[15] Fahme SA, Sieverding M, Abdulrahim S. Sexual and reproductive health of adolescent Syrian refugee girls in Lebanon: a qualitative study of healthcare provider and educator perspectives. Reprod Health. 2021 Jun 6;18(1):113. doi: 10.1186/s12978-021-01170-3 34092236 PMC8183084

[16] Bouclaous C, Haddad I, Alrazim A, Kolanjian H, El Safadi A. Health literacy levels and correlates among refugees in Mount Lebanon. Public Health. 2021 Oct;199:25–31. doi: 10.1016/j.puhe.2021.08.006 34534886

[17] World Health Organization. Global and regional sexually transmitted infection estimates for 2020. Available from https://www.who.int/data/gho/data/themes/topics/global-and-regional-sti-estimates. Accessed on December 18, 2023. World Health Organization 2023.

[18] Kombe Kombe AJ, Li B, Zahid A, Mengist HM, Bounda GA, Zhou Y, et al. Epidemiology and burden of human papillomavirus and related diseases, molecular pathogenesis, and vaccine evaluation. *Front Public Health*. 2021 Jan 20;8:552028. doi: 10.3389/fpubh.2020.552028 33553082 PMC7855977

[19] Smolak A, Chemaitelly H, Hermez JG, Low N, Abu-Raddad LJ. Epidemiology of Chlamydia trachomatis in the Middle East and north Africa: a systematic review, meta-analysis, and meta-regression. *Lancet Glob Health*. 2019 Sep;7(9):e1197–e1225. doi: 10.1016/S2214-109X(19)30279-7 31402004

[20] Mumtaz GR, Chemaitelly H, AlMukdad S, Osman A, Fahme S, Rizk NA, et al. Status of the HIV epidemic in key populations in the Middle East and North Africa: knowns and unknowns. *Lancet HIV*. 2022 Jul;9(7):e506–e516. doi: 10.1016/S2352-3018(22)00093-5 35777412

[21] Chemaitelly H, Majed A, Abu-Hijleh F, Blondeel K, Matsaseng TC, Kiarie J, et al. Global epidemiology of Neisseria gonorrhoeae in infertile populations: systematic review, meta-analysis and metaregression. *Sex Transm Infect*. 2021 Mar;97(2):157–169. doi: 10.1136/sextrans-2020-054515 32423944 PMC7892374

[22] Finan RR, Chemaitelly H, Racoubian E, Aimagambetova G, Almawi WY. Genetic diversity of human papillomavirus (HPV) as specified by the detection method, gender, and year of sampling: a retrospective cross-sectional study. *Arch Gynecol Obstet*. 2023 May;307(5): 1469–1479. doi: 10.1007/s00404-022-06907-4 36624228

[23] Al-Awadhi R, Chehadeh W, Kapila K. Prevalence of human papillomavirus among women with normal cervical cytology in Kuwait. *J Med Virol*. 2011 Mar;83(3):453–60. doi: 10.1002/jmv.21981 21264866

[24] Zheng LL, Chen SF, Yang F, Wang WH, Xu C, Zheng LY. High-risk HPV prevalence and genotype distribution among women in Liaocheng, Shandong Province, China from 2016 to 2022. *Front Public Health*. 2023 Mar 30;11:1145396. doi: 10.3389/fpubh.2023.1145396 37064671 PMC10098111

[25] Malik S, Sah R, Muhammad K, Waheed Y. Tracking HPV Infection, Associated Cancer Development, and Recent Treatment Efforts-A Comprehensive Review. Vaccines (Basel). 2023 Jan 1;11(1):102. doi: 10.3390/vaccines11010102 36679945 PMC9860736

[26] Babi A, Issa T, Issanov A, Akhanova S, Udalova N, Koktova S, et al. Knowledge and attitudes of mothers toward HPV vaccination: A cross-sectional study in Kazakhstan. *Womens Health (Lond)*. 2023 Jan-Dec;19:17455057231172355. doi: 10.1177/17455057231172355 37184051 PMC10192804

[27] Finan RR, Tamim H, Almawi WY. Identification of Chlamydia trachomatis DNA in human papillomavirus (HPV) positive women with normal and abnormal cytology. *Arch Gynecol Obstet*. 2002 Jul;266(3):168–71. doi: 10.1007/s00404-001-0261-8 12197559

[28] López-Corbeto E, González V, Casabona J; Grupo de estudio CT NG. First data of Chlamydia trachomatis and other STI prevalence and co-infections in pregnant women under 25 years in Catalonia, Spain. *Med Clin (Barc)*. 2021 Jan 8;156(1):33–34. doi: 10.1016/j.medcli.2019.12.019 32143940

[29] Kapiga S, Kelly C, Weiss S, Daley T, Peterson L, Leburg C, et al. Risk factors for incidence of sexually transmitted infections among women in South Africa, Tanzania, and Zambia: results from HPTN 055 study. *Sex Transm Dis*. 2009 Apr;36(4):199–206. doi: 10.1097/OLQ.0b013e318191ba01 19265734

[30] Skaletz-Rorowski A, Potthoff A, Nambiar S, Wach J, Kayser A, Kasper A, et al. Age specific evaluation of sexual behavior, STI knowledge and infection among asymptomatic adolescents and young adults. *J Infect Public Health*. 2020 Aug;13(8):1112–1117. doi: 10.1016/j.jiph.2020.04.005 32471797

[31] Adebamowo SN, Ma B, Zella D, Famooto A, Ravel J, Adebamowo C, et al. Mycoplasma hominis and Mycoplasma genitalium in the Vaginal Microbiota and Persistent High-Risk Human Papillomavirus Infection. *Front Public Health*. 2017 Jun 26;5:140. doi: 10.3389/fpubh.2017.00140 28695118 PMC5483445

[32] Almawi WY, Finan RR, Tamim H, Daccache JL, Irani-Hakime N. Differences in the frequency of the C677T mutation in the methylenetetrahydrofolate reductase (MTHFR) gene among the Lebanese population. *Am J Hematol*. 2004 May;76(1):85–7. doi: 10.1002/ajh.20047 15114606

