# Supplementary material for: Estimates of the incidence, prevalence, and factors associated with common sexually transmitted infections among Lebanese women
Source: PLoS One. 2024 Apr 18;19(4):e0301231. doi: 10.1371/journal.pone.0301231 (PMC11025747; doi:10.1371/journal.pone.0301231)
Supplement: S1 Table — (DOCX) [file pone.0301231.s003.docx]

|  | **Item No** | **Recommendation** | **Main Text** |
| --- | --- | --- | --- |
| **Title and abstract** | 1 | (*a*) Indicate the study’s design with a commonly used term in the title or the abstract | Abstract |
|  |  | (*b*) Provide in the abstract an informative and balanced summary of what was done and what was found | Abstract |
| **Introduction** | | |  |
| Background/rationale | 2 | Explain the scientific background and rationale for the investigation being reported | Introduction |
| Objectives | 3 | State specific objectives, including any prespecified hypotheses | Introduction |
| **Methods** | | |  |
| Study design | 4 | Present key elements of study design early in the paper | Subjects and Methods (‘Study subjects’ & ‘Specimens’ collection’) |
| Setting | 5 | Describe the setting, locations, and relevant dates, including periods of recruitment, exposure, follow-up, and data collection | Subjects and Methods |
| Participants | 6 | (*a*) Give the eligibility criteria, and the sources and methods of selection of participants | Subjects and Methods (‘Study subjects’) |
| Variables | 7 | Clearly define all outcomes, exposures, predictors, potential confounders, and effect modifiers. Give diagnostic criteria, if applicable | Subjects and Methods & Table 1 |
| Data sources/ measurement | 8* | For each variable of interest, give sources of data and details of methods of assessment (measurement). Describe comparability of assessment methods if there is more than one group | Subjects and Methods (‘Specimens’ collection’, ‘HPV DNA amplification’, & ‘Statistical analysis’) |
| Bias | 9 | Describe any efforts to address potential sources of bias | Subjects and Methods (‘Statistical analysis’) |
| Study size | 10 | Explain how the study size was arrived at | Subjects and Methods (‘Study subjects’) |
| Quantitative variables | 11 | Explain how quantitative variables were handled in the analyses. If applicable, describe which groupings were chosen and why | Subjects and Methods (‘HPV DNA amplification’ & ‘Statistical analysis’), & Table 1 |
| Statistical methods | 12 | (*a*) Describe all statistical methods, including those used to control for confounding | Subjects and Methods (‘Statistical analysis’) |
|  |  | (*b*) Describe any methods used to examine subgroups and interactions | Subjects and Methods (‘Statistical analysis’) |
|  |  | (*c*) Explain how missing data were addressed | Subjects and Methods (‘Statistical analysis’) |
|  |  | (*d*) If applicable, describe analytical methods taking account of sampling strategy | Not applicable |
|  |  | (*e*) Describe any sensitivity analyses | Not applicable |
| **Results** | | |  |
| Participants | 13* | (a) Report numbers of individuals at each stage of study—eg numbers potentially eligible, examined for eligibility, confirmed eligible, included in the study, completing follow-up, and analysed | Results (‘Study population’) & Table 1 |
|  |  | (b) Give reasons for non-participation at each stage |  |
|  |  | (c) Consider use of a flow diagram |  |
| Descriptive data | 14* | (a) Give characteristics of study participants (eg demographic, clinical, social) and information on exposures and potential confounders | Results (‘Study population’) & Table 1 |
|  |  | (b) Indicate number of participants with missing data for each variable of interest | Table 1 |
| Outcome data | 15* | Report numbers of outcome events or summary measures | Results (‘Active infection prevalence’), Table 1 & Figure 1 |
| Main results | 16 | (*a*) Give unadjusted estimates and, if applicable, confounder-adjusted estimates and their precision (eg, 95% confidence interval). Make clear which confounders were adjusted for and why they were included | Results (‘Active infection prevalence’ & ‘Patterns of active infection’), Table 1 & Figures 1-2 |
|  |  | (*b*) Report category boundaries when continuous variables were categorized | Table 1 |
|  |  | (*c*) If relevant, consider translating estimates of relative risk into absolute risk for a meaningful time period | Not applicable |
| Other analyses | 17 | Report other analyses done—eg analyses of subgroups and interactions, and sensitivity analyses | Not applicable |
| **Discussion** | | |  |
| Key results | 18 | Summarise key results with reference to study objectives | Discussion, paragraphs 1-4 |
| Limitations | 19 | Discuss limitations of the study, taking into account sources of potential bias or imprecision. Discuss both direction and magnitude of any potential bias | Discussion, paragraph 5 |
| Interpretation | 20 | Give a cautious overall interpretation of results considering objectives, limitations, multiplicity of analyses, results from similar studies, and other relevant evidence | Discussion, paragraph 15 |
| Generalisability | 21 | Discuss the generalisability (external validity) of the study results | Discussion, paragraph 6 |
| **Other information** | | |  |
| Funding | 22 | Give the source of funding and the role of the funders for the present study and, if applicable, for the original study on which the present article is based | Funding |
