# Supplementary material for: Estimates of the incidence, prevalence, and factors associated with common sexually transmitted infections among Lebanese women
Source: PLoS One. 2024 Apr 18;19(4):e0301231. doi: 10.1371/journal.pone.0301231 (PMC11025747; doi:10.1371/journal.pone.0301231)
Supplement: S2 Table — (DOCX) [file pone.0301231.s004.docx]

**Table S2. Associations with active infection with any of *Neisseria gonorrhoeae*, *Chlamydia trachomatis*, *Trichomonas vaginalis*, and *Treponema pallidum*.**

| **Characteristics** | **Tested** | **PCR-positive** | | **Univariable regression analysis** | | **Multivariable regression analysis** | |
| --- | --- | --- | --- | --- | --- | --- | --- |
|  | N | N (%) | p-value | OR (95% CI) | F test p-value | AOR (95% CI) | p-value^*^ |
| Age—years |  |  | 0.831 |  | 0.821 |  |  |
| 20-29 years | 100 | 2 (2.0) |  | 1.00 |  | 1.00 |  |
| 30-39 years | 191 | 6 (3.1) |  | 1.59 (0.31-8.02) |  | 1.65 (0.31-8.65) | 0.553 |
| 40+ years | 60 | 2 (3.3) |  | 1.69 (0.23-12.32) |  | 1.77 (0.23-13.34) | 0.580 |
| Smoking |  |  | 0.926 |  | 0.926 |  |  |
| No | 241 | 7 (2.9) |  | 1.00 |  | 1.00 |  |
| Yes | 110 | 3 (2.7) |  | 0.94 (0.24-3.69) |  | 0.82 (0.19-3.45) | 0.782 |
| Marital |  |  | 0.911 |  | 0.910 |  |  |
| Married | 240 | 7 (2.9) |  | 1.00 |  | 1.00 |  |
| Single/Divorced/Separated | 111 | 3 (2.7) |  | 0.92 (0.23-3.64) |  | 0.87 (0.20-3.84) | 0.854 |
| Numbers of partners |  |  | 0.571 |  | 0.574 |  |  |
| 0-1 partner | 206 | 5 (2.4) |  | 1.00 |  | 1.00 |  |
| 2+ partners | 145 | 5 (3.4) |  | 1.44 (0.41-5.05) |  | 1.68 (0.42-6.71) | 0.461 |

**AOR**, adjusted odds ratio; **CI**, confidence interval; **OR**, odds ratio.

^*^Covariates with p-value ≤0.05 in the multivariable analysis were considered as showing strong evidence for an association with active infection.
