# Supplementary material for: Estimates of the incidence, prevalence, and factors associated with common sexually transmitted infections among Lebanese women
Source: PLoS One. 2024 Apr 18;19(4):e0301231. doi: 10.1371/journal.pone.0301231 (PMC11025747; doi:10.1371/journal.pone.0301231)
Supplement: S3 Table — (DOCX) [file pone.0301231.s005.docx]

**Table S3. Associations with active infection with human papillomavirus (HPV).**

| **Characteristics** | **Tested** | **PCR-positive** | | **Univariable regression analysis** | | **Multivariable regression analysis** | |
| --- | --- | --- | --- | --- | --- | --- | --- |
|  | N | N (%) | p-value | OR (95% CI) | F test p-value | AOR (95% CI) | p-value^*^ |
| **High-risk HPV** |  |  |  |  |  |  |  |
| Age—years |  |  | 0.007 |  | 0.004 |  |  |
| 20-29 years | 100 | 7 (7.0) |  | 1.00 |  | 1.00 |  |
| 30-39 years | 191 | 40 (20.9) |  | 3.52 (1.51-8.18) |  | 3.49 (1.48-8.22) | 0.004 |
| 40+ years | 60 | 8 (13.3) |  | 2.04 (0.70-5.96) |  | 2.05 (0.69-6.06) | 0.193 |
| Smoking |  |  | 0.479 |  | 0.474 |  |  |
| No | 241 | 40 (16.6) |  | 1.00 |  | 1.00 |  |
| Yes | 110 | 15 (13.6) |  | 0.79 (0.41-1.51) |  | 0.82 (0.42-1.63) | 0.576 |
| Marital |  |  | 0.450 |  | 0.445 |  |  |
| Married | 240 | 40 (16.7) |  | 1.00 |  | 1.00 |  |
| Single/Divorced/Separated | 111 | 15 (13.5) |  | 0.78 (0.41-1.48) |  | 0.97 (0.48-1.95) | 0.934 |
| Numbers of partners |  |  | 0.608 |  | 0.607 |  |  |
| 0-1 partner | 206 | 34 (16.5) |  | 1.00 |  | 1.00 |  |
| 2+ partners | 145 | 21 (14.5) |  | 0.86 (0.47-1.55) |  | 1.03 (0.53-1.99) | 0.934 |
| **Low-risk HPV** |  |  |  |  |  |  |  |
| Age—years |  |  | 0.128 |  | 0.109 |  |  |
| 20-29 years | 100 | 7 (7.0) |  | 1.00 |  | 1.00 |  |
| 30-39 years | 191 | 29 (15.2) |  | 2.38 (1.00-5.64) |  | 2.54 (1.05-6.15) | 0.039 |
| 40+ years | 60 | 7 (11.7) |  | 1.75 (0.58-5.27) |  | 1.91 (0.62-5.85) | 0.257 |
| Smoking |  |  | 0.605 |  | 0.601 |  |  |
| No | 241 | 31 (12.9) |  | 1.00 |  | 1.00 |  |
| Yes | 110 | 12 (10.9) |  | 0.83 (0.41-1.68) |  | 0.86 (0.40-1.81) | 0.684 |
| Marital |  |  | 0.624 |  | 0.626 |  |  |
| Married | 240 | 28 (11.7) |  | 1.00 |  | 1.00 |  |
| Single/Divorced/Separated | 111 | 15 (13.5) |  | 1.18 (0.60-2.32) |  | 1.54 (0.74-3.22) | 0.253 |
| Numbers of partners |  |  | 0.560 |  | 0.558 |  |  |
| 0-1 partner | 206 | 27 (13.1) |  | 1.00 |  | 1.00 |  |
| 2+ partners | 145 | 16 (11.0) |  | 0.82 (0.43-1.59) |  | 0.82 (0.39-1.71) | 0.593 |

**AOR**, adjusted odds ratio; **CI**, confidence interval; **OR**, odds ratio.

^*^Covariates with p-value ≤0.05 in the multivariable analysis were considered as showing strong evidence for an association with active infection.
