# Supplementary material for: Estimates of the incidence, prevalence, and factors associated with common sexually transmitted infections among Lebanese women
Source: PLoS One. 2024 Apr 18;19(4):e0301231. doi: 10.1371/journal.pone.0301231 (PMC11025747; doi:10.1371/journal.pone.0301231)
Supplement: S4 Table — (DOCX) [file pone.0301231.s006.docx]

**Table S4. Associations with active infection with *Mycoplasma hominis* and *Mycoplasma genitalium*.**

| **Characteristics** | **Tested** | **PCR-positive** | | **Univariable regression analysis** | | **Multivariable regression analysis** | |
| --- | --- | --- | --- | --- | --- | --- | --- |
|  | N | N (%) | p-value | OR (95% CI) | F test p-value | AOR (95% CI) | p-value^*^ |
| ***Mycoplasma hominis*** |  |  |  |  |  |  |  |
| Age—years |  |  | 0.665 |  | 0.004 |  |  |
| 20-29 years | 100 | 20 (20.0) |  | 1.00 |  | 1.00 |  |
| 30-39 years | 191 | 42 (22.0) |  | 1.13 (0.62-2.05) |  | 1.29 (0.69-2.41) | 0.418 |
| 40+ years | 60 | 10 (16.7) |  | 0.80 (0.35-1.85) |  | 0.87 (0.37-2.04) | 0.743 |
| Smoking |  |  | 0.034 |  | 0.035 |  |  |
| No | 241 | 42 (17.4) |  | 1.00 |  | 1.00 |  |
| Yes | 110 | 30 (27.3) |  | 1.78 (1.04-3.04) |  | 1.59 (0.90-2.80) | 0.111 |
| Marital |  |  | 0.137 |  | 0.139 |  |  |
| Married | 240 | 44 (18.3) |  | 1.00 |  | 1.00 |  |
| Single/Divorced/Separated | 111 | 28 (25.2) |  | 1.50 (0.88-2.58) |  | 1.30 (0.72-2.35) | 0.391 |
| Numbers of partners |  |  | 0.051 |  | 0.053 |  |  |
| 0-1 partner | 206 | 35 (17.0) |  | 1.00 |  | 1.00 |  |
| 2+ partners | 145 | 37 (25.5) |  | 1.67 (0.99-2.82) |  | 1.37 (0.76-2.47) | 0.289 |
| ***Mycoplasma genitalium*** |  |  |  |  |  |  |  |
| Age—years |  |  | 0.632 |  | 0.607 |  |  |
| 20-29 years | 100 | 1 (1.0) |  | 1.00 |  | 1.00 |  |
| 30-39 years | 191 | 5 (2.6) |  | 2.66 (0.31-23.10) |  | 2.03 (0.23-18.35) | 0.527 |
| 40+ years | 60 | 1 (1.7) |  | 1.68 (0.10-27.33) |  | 1.19 (0.07-20.41) | 0.902 |
| Smoking |  |  | 0.507 |  | 0.518 |  |  |
| No | 241 | 4 (1.7) |  | 1.00 |  | 1.00 |  |
| Yes | 110 | 3 (2.7) |  | 1.66 (0.37-7.55) |  | 2.68 (0.55-12.94) | 0.220 |
| Marital |  |  | 0.319 |  | 0.285 |  |  |
| Married | 240 | 6 (2.5) |  | 1.00 |  | 1.00 |  |
| Single/Divorced/Separated | 111 | 1 (0.9) |  | 0.35 (0.04-2.98) |  | 0.59 (0.06-5.52) | 0.640 |
| Numbers of partners |  |  | 0.142 |  | 0.117 |  |  |
| 0-1 partner | 206 | 6 (2.9) |  | 1.00 |  | 1.00 |  |
| 2+ partners | 145 | 1 (0.7) |  | 0.23 (0.03-1.94) |  | 0.21 (0.02-2.07) | 0.183 |

**AOR**, adjusted odds ratio; **CI**, confidence interval; **OR**, odds ratio.

^*^Covariates with p-value ≤0.05 in the multivariable analysis were considered as showing strong evidence for an association with active infection.
