# Supplementary material for: Estimates of the incidence, prevalence, and factors associated with common sexually transmitted infections among Lebanese women
Source: PLoS One. 2024 Apr 18;19(4):e0301231. doi: 10.1371/journal.pone.0301231 (PMC11025747; doi:10.1371/journal.pone.0301231)
Supplement: Attachment — Submitted filename: Review.pdf [file pone.0301231.s008.pdf]

I consider the paper relevant but need some explanation about the topics below.

The article does not clarify whether the tested women attended the gynecology outpatient clinic with complaints (symptoms or clinical signs) or just for routine gynecological check-ups (preventive). There is also no confirmation whether among the examined women there pregnant women and any reference to the gestational period of these ones were. This fact could impact the discovery of STIs by the research.

I suggest to the authors that they separate the group of women without sexual partners from those with one partner and those with two or more sexual partners. Otherwise, it is necessary to detail in the materials and methods because the separation was not made. I also understand it is necessary to establish unequivocally the period reflected by the number of sexual partners, as only using the expression " lifetime sexual partners " in the discussion is not sufficient.

I am curious about the information regarding whether there is a statistically significant difference between the median age of the participants and a positive result for Neisseria gonorrhoeae, Chlamydia trachomatis, Trichomonas vaginalis, and Treponema pallidum.
