# Supplementary material for: Estimates of the incidence, prevalence, and factors associated with common sexually transmitted infections among Lebanese women
Source: PLoS One. 2024 Apr 18;19(4):e0301231. doi: 10.1371/journal.pone.0301231 (PMC11025747; doi:10.1371/journal.pone.0301231)
Supplement: Attachment — Submitted filename: Reviewer Comments.pdf [file pone.0301231.s007.pdf]

PLOS ONE

Estimates of the incidence, prevalence, and factors associated with common sexually transmitted infections among Lebanese women

--Manuscript Draft--

**Manuscript Number:**	PONE-D-24-03237
**Article Type:**	Research Article
**Full Title:**	Estimates of the incidence, prevalence, and factors associated with common sexually transmitted infections among Lebanese women
**Short Title:**	Sexually Transmitted Infections Among Women
**Corresponding Author:**	Wassim Y Almawi<br>University of Tunis El Manar: Universite de Tunis El Manar<br>Tunis, TUNISIA
**Keywords:**	Human papillomavirus; Mycobacteria; Sexually transmitted infections
**Abstract:**	Background. We analyzed the prevalence of active infection with common curable sexually transmitted infections (STIs) including N. gonorrhea,C. trachomatis, T. vaginalis, and T. pallidum, as well as active infection with HPV, herpes simplex virus types I (HSV-1) and II (HSV-2), M. hominis,M. genitalium, C. albicans, and Ureaplasma in 351 Lebanese women.<br>Methods. A cross-sectional study, involving 351 sexually active women, 40 years or younger, who were recruited from outpatient Obstetrics and Gynecology clinic attendees between September 2016 and November 2017.<br>Results. The prevalence of active infection was low at 0.3% for N. gonorrhea, 0.6% for HSV-2, 2.8% for C. trachomatis, and 2.9% for any curable STIs. Prevalence of active HPV infection was high assessed at 15.7% for high-risk and 12.2% for low-risk genotypes. Furthermore, the prevalence was 2.0% for M. genitalium, 6.8% for ureaplasma, 13.7% for Candida albicans, and 20.5% for M. hominis. No active infections with T. vaginalis, T. pallidum, or HSV-1 were observed. Significant age differences were noted in the prevalence of high-risk and low-risk HPV genotypes, but no such differences were noted in the prevalence of other infections. No appreciable variations were identified in the prevalence of key STIs based on smoking, marital status, or the number of sexual partners.<br>Conclusions. The study documented active infection with substantial prevalence for multiple STIs among women attending outpatient gynecology and obstetrics clinics in Lebanon. These findings underscore the importance of strengthening STI surveillance, linkage to care, and prevention interventions in reducing STI incidence among women.
**Order of Authors:**	Hiam Chemaitelly
	Ramzi R. Finan
	Eddie Racoubian
	Gulzhanat Aimagambetova
	Wassim Y Almawi
**Additional Information:**	
**Question**	**Response**
**Financial Disclosure**<br><br>Enter a financial disclosure statement that describes the sources of funding for the work included in this submission. Review the submission guidelines for detailed requirements. View published research articles from *PLOS ONE* for specific examples.	The author(s) received no specific funding for this work.

This statement is required for submission and **will appear in the published article** if the submission is accepted. Please make sure it is accurate.<br><br>**Funded studies**<br>Enter a statement with the following details:<br>• Initials of the authors who received each award<br>• Grant numbers awarded to each author<br>• The full name of each funder<br>• URL of each funder website<br>• Did the sponsors or funders play any role in the study design, data collection and analysis, decision to publish, or preparation of the manuscript?<br><br>Did you receive funding for this work?	
**Competing Interests**<br><br>Use the instructions below to enter a competing interest statement for this submission. On behalf of all authors, disclose any competing interests that could be perceived to bias this work—acknowledging all financial support and any other relevant financial or non-financial competing interests.<br><br>This statement is required for submission and **will appear in the published article** if the submission is accepted. Please make sure it is accurate and that any funding sources listed in your Funding Information later in the submission form are also declared in your Financial Disclosure statement.<br><br>View published research articles from *PLOS ONE* for specific examples.	The authors have declared that no competing interests exist.

**NO authors have competing interests**<br><br>Enter: *The authors have declared that no competing interests exist.*<br><br>**Authors with competing interests**<br><br>Enter competing interest details beginning with this statement:<br><br>*I have read the journal's policy and the authors of this manuscript have the following competing interests: [insert competing interests here]*<br><br>* typeset	
**Ethics Statement**<br><br>Enter an ethics statement for this submission. This statement is required if the study involved:<br><br>• Human participants<br>• Human specimens or tissue<br>• Vertebrate animals or cephalopods<br>• Vertebrate embryos or tissues<br>• Field research<br><br>Write "N/A" if the submission does not require an ethics statement.<br><br>General guidance is provided below. Consult the submission guidelines for detailed instructions. **Make sure that all information entered here is included in the Methods section of the manuscript.**	This study was performed in line with the principles of the Declaration of Helsinki. Approval was granted by the Research and Ethics Committee of St. Marc Medical Center (Number, SMMC-2019-0047), granted on October 17, 2019. This study was reported according to the Strengthening the Reporting of Observational Studies in Epidemiology (STROBE) guidelines (Table S1 in Supplementary Appendix).

Powered by Editorial Manager® and ProduXion Manager® from Aries Systems Corporation

**Format for specific study types**<br><br>**Human Subject Research (involving human participants and/or tissue)**<br>• Give the name of the institutional review board or ethics committee that approved the study<br>• Include the approval number and/or a statement indicating approval of this research<br>• Indicate the form of consent obtained (written/oral) or the reason that consent was not obtained (e.g. the data were analyzed anonymously)<br><br>**Animal Research (involving vertebrate animals, embryos or tissues)**<br>• Provide the name of the Institutional Animal Care and Use Committee (IACUC) or other relevant ethics board that reviewed the study protocol, and indicate whether they approved this research or granted a formal waiver of ethical approval<br>• Include an approval number if one was obtained<br>• If the study involved *non-human primates*, add *additional details* about animal welfare and steps taken to ameliorate suffering<br>• If anesthesia, euthanasia, or any kind of animal sacrifice is part of the study, include briefly which substances and/or methods were applied<br><br>**Field Research**<br><br>Include the following details if this study involves the collection of plant, animal, or other materials from a natural setting:<br>• Field permit number<br>• Name of the institution or relevant body that granted permission	
**Data Availability**<br><br>Authors are required to make all data underlying the findings described fully available, without restriction, and from the time of publication. PLOS allows rare exceptions to address legal and ethical concerns. See the PLOS Data Policy and FAQ for detailed information.	No - some restrictions will apply

Powered by Editorial Manager® and ProduXion Manager® from Aries Systems Corporation

A Data Availability Statement describing where the data can be found is required at submission. Your answers to this question constitute the Data Availability Statement and **will be published in the article**, if accepted.<br><br>**Important:** Stating 'data available on request from the author' is not sufficient. If your data are only available upon request, select 'No' for the first question and explain your exceptional situation in the text box.<br><br>Do the authors confirm that all data underlying the findings described in their manuscript are fully available without restriction?	
**Describe where the data may be found in full sentences. If you are copying our sample text, replace any instances of XXX with the appropriate details.**<br><br>• If the data are **held or will be held in a public repository**, include URLs, accession numbers or DOIs. If this information will only be available after acceptance, indicate this by ticking the box below. For example: *All XXX files are available from the XXX database (accession number(s) XXX, XXX.).*<br>• If the data are all contained **within the manuscript and/or Supporting Information files**, enter the following: *All relevant data are within the manuscript and its Supporting Information files.*<br>• If neither of these applies but you are able to provide **details of access elsewhere**, with or without limitations, please do so. For example:<br><br>*Data cannot be shared publicly because of [XXX]. Data are available from the XXX Institutional Data Access / Ethics Committee (contact via XXX) for researchers who meet the criteria for access to confidential data.*<br><br>*The data underlying the results presented in the study are available from (include the name of the third party*	The datasets generated and/or analyzed during the current study are available on reasonable request from the principal investigator (WY Almawi) upon request.

and contact information or URL).<br>• This text is appropriate if the data are owned by a third party and authors do not have permission to share the data.<br><br>* typeset	
Additional data availability information:	

Manuscript

Click here to access/download;Manuscript;PLoS One HPV STD3 main text.docx

Estimates of the incidence, prevalence, and factors associated with common sexually transmitted infections among Lebanese women

Hiam Chemaitelly [1,2], Ramzi R. Finan [3], Eddie Racoubian [4], Gulzhanat Aimagambetova [5], Wassim Y. Almawi [6,7]

1 Infectious Disease Epidemiology Group, Weill Cornell Medicine-Qatar, Cornell University, Doha, Qatar

2 Department of Population Health Sciences, Weill Cornell Medicine, Cornell University, New York, USA

3 Department of Obstetrics and Gynecology, Hôtel Dieu de France, CHU Université St. Joseph, Beirut, Lebanon,

4 St. March Medical and Diagnostic Center, Beirut, Lebanon

5 Department of Surgery, School of Medicine, Nazarbayev University, Astana, Kazakhstan

6 Department of Biological Sciences, Brock University, St. Catharines, Ontario, Canada

7 Faculty of Sciences, El Manar University, Tunis, Tunisia

Running Title: Sexually Transmitted Infections Among Women

Corresponding Author: Wassim Y. Almawi, Ph.D. Faculté des Sciences de Tunis, Université de Tunis El Manar, Campus Universitaire El-Manar, 2092 El Manar, Tunis, Tunisia. Tel +216 71 872 600, Fax : +216 71 871 666. E-mail. wassim.almawi@outlook.com. **ORCID**: 0000-0003-1633-9757.

Abstract

Background. We analyzed the prevalence of active infection with common curable sexually transmitted infections (STIs) including *N. gonorrhea, C. trachomatis*, *T. vaginalis,* and *T. pallidum*, as well as active infection with HPV, herpes simplex virus types I (HSV-1) and II (HSV-2), *M. hominis, M. genitalium*, *C. albicans*, and Ureaplasma in 351 Lebanese women.

Methods. A cross-sectional study, involving 351 sexually active women, 40 years or younger, who were recruited from outpatient Obstetrics and Gynecology clinic attendees between September 2016 and November 2017.

Results. The prevalence of active infection was low at 0.3% for *N. gonorrhea*, 0.6% for HSV-2, 2.8% for *C. trachomatis*, and 2.9% for any curable STIs. Prevalence of active HPV infection was high assessed at 15.7% for high-risk and 12.2% for low-risk genotypes. Furthermore, the prevalence was 2.0% for *M. genitalium*, 6.8% for ureaplasma, 13.7% for *Candida albicans,* and 20.5% for *M. hominis*. No active infections with *T. vaginalis*, *T. pallidum*, or HSV-1 were observed. Significant age differences were noted in the prevalence of high-risk and low-risk HPV genotypes, but no such differences were noted in the prevalence of other infections. No appreciable variations were identified in the prevalence of key STIs based on smoking, marital status, or the number of sexual partners.

Conclusions. The study documented active infection with substantial prevalence for multiple STIs among women attending outpatient gynecology and obstetrics clinics in Lebanon. These findings underscore the importance of strengthening STI surveillance, linkage to care, and prevention interventions in reducing STI incidence among women.

Key Words: Human papillomavirus; Mycobacteria; Sexually transmitted infections

Introduction

Sexually transmitted infections (STIs) remain a global pervasive public health challenge, with women across wide age groups, and socioeconomic and geographical backgrounds, bearing a significant portion of this burden [1,2]. STIs encompass an array of bacterial, viral, and parasitic infections that are transmitted through sexual contact, and range from the common chlamydia and gonorrhea to the more serious and likely life-threatening infections such as HIV/AIDS [2,3]. Several factors are associated with the persistence of STIs, which include changes in sexual behavior, lack of sufficient awareness, evolving pathogens, inadequate access to healthcare, and stigma [4,5]. STIs differ in their scope, characteristics, prognosis, and consequences, which can vary from localized inflammatory disorder to cancer, and even death [4,6], thus necessitating comprehensive STI investigation and testing in women at a young age, and periodically thereafter. Infection with STIs can result in a range of associated complications if left untreated [7,8]. These include cervical cancer, infertility, chronic pain, and heightened neonatal morbidity and mortality [2,9]. Accurate and timely detection of active STIs is crucial for the affected individual and public health management [9,10]. The advent of DNA/RNA-based PCR has allowed for highly sensitive and specific detection of STI pathogens in different sample types, including blood, urine, and vaginal discharges [9,11]. Despite the availability of ultra-sensitive diagnostic tests for STI causative agents, the prevalence of STIs remains a public health concern, both in developed, developing, and under-developed countries [2,6].

This study investigated the prevalence of active infection with *N. gonorrhea, C. trachomatis*, *T. vaginalis*, *T. pallidum,* Human papillomavirus (HPV), herpes simplex virus types I (HSV-1) and II (HSV-2), *M. hominis*, *M. genitalium*, *C. albicans*, and ureaplasma in 351 Lebanese women attending outpatient obstetrics and gynecology (OB/GYN) clinics, of whom 291 were 40 years or younger. The study further explored the variations in prevalence by age, smoking status, marital status, and number of sexual partners.

Subjects and Methods

Study subjects. The study participants comprised 351 women attending the outpatient OB/GYN clinics at Hôtel-Dieu de France Hospital and St. Marc Medical & Diagnostic Center in Greater Beirut. This study was performed in line with the principles of the Declaration of Helsinki. Approval was granted by the Research and Ethics Committee of St. Marc Medical Center (Number, SMMC-2019-0047), granted on October 17, 2019. This study was reported according to the Strengthening the Reporting of Observational Studies in Epidemiology (STROBE) guidelines (Table S1 in Supplementary Appendix). Data included in this retrospective study were obtained from the medical records for research purposes and were accessed between November 1, 2019, and November 30, 2020. While the patients' socio-demographic and risk behavior data were retrieved through a review of medical charts, the identity of the individual participants was anonymized throughout the study.

Specimens' collection. Endocervical specimens and first-catch urine specimens were then collected and processed within 4 hours of collection. Swabs were collected by a gynecologist using speculum-assisted Spatula of Ayre after removing the mucus and were placed in a balanced saline solution. Total genomic DNA was extracted by the mini-spin column method (Qiagen, Hilden, Germany). DNA from urine specimens was extracted by the Roche Amplicor STD specimen preparation kit (Roche Diagnostics, Mannheim, Germany), according to the manufacturer's specifications.

HPV DNA amplification. HPV testing on endocervical DNA samples was done by nested PCR using MY09 and MY11 external primers and GP05+/GP06+ internal primers, as shown elsewhere (Finan et al., 2006). HPV genotypes were detected by HPV Quant-21® (DNA Technology, Moscow, Russian Federation), and were grouped into low-risk (LR) (HPV6, 11, 42, 43, 44, and 70), and high-risk (HR; HPV16, 18, 26, 31, 33, 35, 39, 45, 51, 52, 53, 56, 58, 59, 66, 68, 73, and 82). Detection of other STIs was also done using a specific DNA Technology Detection kit (DNA Technology).

Statistical analysis. Participant characteristics were summarized using both frequency distributions and measures of central tendency. In cases where specific variables had missing values (constituting ≤2.2% of the dataset), these values were imputed using the median of

observed outcomes for individuals with complete data. Descriptive statistics were performed on all testing samples, and the prevalence of active infections, along with the corresponding 95% confidence intervals (CI), were estimated. Stratified analyses were conducted to investigate the distribution of active infections across various factors, including age, smoking status, marital status, and number of sexual partnerships. Chi-square tests were performed to assess the association of each covariate with active infection. A p-value below 0.05 indicated a strong association with active infection. Due to the limited number of PCR-positive samples for *N. gonorrhea*, *C. trachomatis*, *T. vaginalis*, and *T. pallidum* (syphilis), these infections were combined to define the presence of any curable STI. To gain deeper insights into the determinants of active infection with key STIs, exploratory univariate and multivariate regression analyses were performed. However, these analyses had insufficient statistical power to detect statistical significance. All statistical analyses were conducted using Stata/SE version 17.0 (Stata Corporation, College Station, TX, USA).

Ethical statement. This study was performed in line with the principles of the Declaration of Helsinki. Approval was granted by the Research and Ethics Committee of St. Marc Medical Center (Number, SMMC-2019-0047), granted on October 17, 2019. This study was reported according to the Strengthening the Reporting of Observational Studies in Epidemiology (STROBE) guidelines (Table S1 in Supplementary Appendix).

Results

Study population. The study included 351 women who underwent testing for an active STI infection. Table 1 shows the characteristics of the study participants. The median age was 33.0 years, with an interquartile range (IQR) of 29.0-37.0 years. Approximately 70% were married, and the rest were single, divorced, or separated. A third of women identified as smokers. About two-thirds reported up to one sexual partner, whereas 15.9% had two sexual partners, and 25.4% had three or more sexual partners.

Active infection prevalence. Figure 1 shows the prevalence of active infections as identified through PCR among study participants. Prevalence was nil (95% CI: 0.0-1.0%) for HSV-1 and low at 0.6% (95% CI: 0.1-2.0%) for HSV-2, indicating minimal instances of active viral shedding. For

curable bacterial STIs, the prevalence was 0.3% (95% CI: 0.0-1.6%) for *N. gonorrhea*, 2.8% (95% CI: 1.4-5.2%) for *C. trachomatis*, and nil (95% CI: 0.0-1.0%) for each of *T. vaginalis* and *T. pallidum*. Prevalence was 2.9% (95% CI: 1.4-5.2%) for any curable STI. Active HPV infection was common with a prevalence of 15.7% (95% CI: 12.0-19.9%) for high-risk genotypes and 12.2% (95% CI: 9.0-16.1%) for low-risk genotypes. Prevalence was 2.0% (95% CI: 0.8-4.1%) for *M. genitalium*, 6.8% (95% CI: 4.4-10.0%) for Ureaplasma, 13.7% (95% CI: 10.3-17.7%) for *Candida albicans*, and 20.5% (95% CI: 16.4-25.1%) for *M. hominis*.

Patterns of active infection. Figure 2 illustrates the distribution of active infection prevalence by age, smoking status, marital status, and number of sexual partners. Detailed statistical associations are provided in Tables S2-S4 in the Supplementary Appendix. Overall, there were negligible variations in prevalence across age groups for *M. genitalium* and for any curable STI, with prevalence ranging from 1.0-2.6% (p-value: 0.632) and 2.0-3.3% (p-value: 0.831), respectively (Figure 2A). A similar pattern was observed for *M. hominis* although prevalence ranged from 16.7-22.0% (p = 0.665). Meanwhile, significant age differences were noted in the prevalence of high-risk HPV. Prevalence was lowest at 7.0% in women aged 20-29 years and highest at 20.9% in women aged 30-39 years (p: value 0.007). Prevalence of low-risk HPV also varied ranging from 7.0% in women aged 20-29 years to 15.2% in those aged 30-39 years (p-value: 0.128).

No appreciable differences were observed in the prevalence of key STIs by smoking status (Figure 2B), except for *M. hominis* where the prevalence was 27.3% among smokers compared to 17.4% among non-smokers (p-value: 0.034). Negligible differences in prevalence were also noted by marital status across all STIs (Figure 2C), despite the prevalence of *M. hominis* being much higher in single, divorced, and separated women (25.2%) compared to married women (18.3%). Minimal variation in the prevalence of key STIs was also found for women reporting up to one sexual partner compared to those reporting two or more sexual partners (Figure 2D). Of note, the prevalence of *M. hominis* was substantially higher in the latter (25.5%) compared to the former group (17.0%; p-value: 0.051).

Discussion

Our assessment of active STI infections in a group of women attending OB/GYN clinics revealed prevalence levels for common STIs that align with global estimates. These prevalence levels are however higher than expected, given the generally conservative sexual norms in the Middle East and North Africa (MENA) region. Specifically, the prevalence of *N. gonorrhoeae* and *C. trachomatis* were 0.3% and 2.8%, respectively, within the CIs of the global prevalences for these infections estimated by the WHO at 0.8% and 3.2%, respectively [12]. The prevalences of high-risk (15.7%) and low-risk (12.2%) HPV genotypes were also considerable, comparable to levels observed in Asia, but lower than levels observed in developed countries such as Europe and North America where routine screening for HPV is implemented [13].

The higher-than-expected prevalences identified among women may not necessarily imply increased engagement in sexual risk behaviors. Instead, these findings underscore the lack of sexual health services and the limited capacity for STI prevention and treatment in Lebanon, and the broader MENA region [14]. Socio-cultural sensitivities, constrained resources, and competing national health priorities have contributed to STIs being neglected in research and surveillance, as well as on national public health agendas [14,15]. Consequently, infections often go undetected, persisting for prolonged periods, and heightening the potential for infection circulation within the population [14]. This situation significantly elevates the risk of future health complications among women [14,16]. The minimal variations in active infection levels by smoking status, marital status, and the number of lifetime sexual partners affirm the notion that insufficient access to STI services is the primary factor contributing to the higher-than-expected prevalence levels.

Our findings showed variability in HR and LR HPV across age groups, with the most elevated prevalences found among women aged 30-39 years [17]. This pattern could signify an increased likelihood of virus exposure over time. However, it may also be influenced by a survival bias, where women in older age groups, having a greater chance of progressing to cervical cancer, are less likely to have survived the complications associated with HPV. These findings are consistent with existing literature indicating the highest prevalence of HPV in this age group, as well as the increased incidence of cervical cancer among older women [18,19]. Consequently, there is a pressing need to integrate HPV screening into routine assessments for women of reproductive age in Lebanon or, at the very least, to enhance awareness about the significance of HPV screening among women, akin to the campaigns established for breast cancer awareness.

Co-infections of HPV-infected women with other STIs, namely Chlamydia, Mycobacteria, and HIV, were linked to HPV persistence, and increased cervical neoplasia risk [4,20]. Insofar as they were reported for different ethnic groups and pathophysiological conditions [3,21], this suggested a geographical pattern of co-infection [10,22], likely linked with socioeconomic and education status and inadequate access to healthcare facilities [5,7,23]. The majority of women with co-infections were positive for HPV (8.6%) as part of double infection with mostly *M. hominis*, which is attributed to the high prevalence of these two STIs globally. This was in line with findings on Nigerian women, in which persistent *M. hominis* infection was linked with HPV positivity, without establishing a clear cause-and-effect link [24]. Other coinfections included mycoplasma with chlamydia and candida, which were not as pronounced as HPV coinfections, in agreement with a recent German study [23]. However, co-infection prevalence was independently associated with smoking and having multiple sexual partners [4,7].

Strengths and Limitations

This study has limitations. The term “co-infection” was based on the detection of nucleic acid material without indication of the actual disease status. Women were recruited from outpatient clinics in a hospital located in the capital city, and therefore our findings may not be generalizable to the broader female population in the country. The number of identified positive

cases was relatively small hindering the conduct of meaningful regression analyses. Despite these shortcomings, this study provided evidence of the current prevalence of a broad spectrum of infections among women in the general population, shedding light on a neglected disease burden.

Conclusions

In conclusion, our study revealed the higher-than-expected prevalence of STIs among women attending OB/GYN clinics in the MENA region, emphasizing the significance of reinforcing STI surveillance to develop a better understanding of the burden of these infections nationwide and monitor infection trends over time [17]. Our findings also underscore the imperative for targeted prevention interventions, including HPV immunization (Babi et al., 2023), enhanced access to sexual health services, integration of STIs into routine screening protocols, and improved linkage to care. Implementing these strategies is essential to reducing STI incidence and mitigating their adverse impact on women's health, social well-being, and economic welfare.

Acknowledgments:

HC is grateful for institutional salary support from the Biomedical Research Program and the Biostatistics, Epidemiology, and Biomathematics Research Core, both at Weill Cornell Medicine-Qatar.

Funding Statement

This work did not receive support from government or non-government agencies

Data Availability

The datasets generated and/or analyzed during the current study are available on reasonable request from the principal investigator (WY Almawi) upon request.

References

1. Mohammed H, Hughes G, Fenton KA. Surveillance systems for sexually transmitted infections: a global review. *Curr Opin Infect Dis*. 2016 Feb;29(1):64-9. doi: 10.1097/QCO.0000000000000235.
2. Unemo M, Bradshaw CS, Hocking JS, de Vries HJC, Francis SC, Mabey D, et al. Sexually transmitted infections: challenges ahead. *Lancet Infect Dis*. 2017 Aug;17(8):e235-e279. doi: 10.1016/S1473-3099(17)30310-9.
3. Menezes LJ, Pokharel U, Sudenga SL, Botha MH, Zeier M, Abrahamsen ME, et al. Patterns of prevalent HPV and STI co-infections and associated factors among HIV-negative young Western Cape, South African women: the EVRI trial. *Sex Transm Infect*. 2018 Feb;94(1):55-61. doi: 10.1136/sextrans-2016-053046.
4. Kops NL, Bessel M, Horvath JDC, Domingues C, de Souza FMA, Benzaken AS, et al. Factors associated with HPV and other self-reported STI coinfections among sexually active Brazilian young adults: cross-sectional nationwide study. *BMJ Open*. 2019 Jun 21;9(6): e027438. doi: 10.1136/bmjopen-2018-027438.
5. Menezes ME, Silver EJ, Goldstein DY, Collins-Ogle MD, Fox AS, Coupey SM. Prevalence and Factors Associated With Mycoplasma genitalium Infection in At-Risk Female Adolescents in Bronx County, New York. *Sex Transm Dis*. 2023 Oct 1;50(10):635-641. doi: 10.1097/OLQ.0000000000001840.
6. Newman L, Rowley J, Vander Hoorn S, Wijesooriya NS, Unemo M, Low N, et al. Global Estimates of the prevalence and incidence of four curable sexually transmitted infections in 2012 based on systematic review and global reporting. *PLoS One*. 2015 Dec 8;10(12): e0143304. doi: 10.1371/journal.pone.0143304.
7. Finan RR, Musharrafieh U, Almawi WY. Detection of Chlamydia trachomatis and herpes simplex virus type 1 or 2 in cervical samples in human papillomavirus (HPV)-positive and HPV-negative women. *Clin Microbiol Infect*. 2006 Sep;12(9):927-30. doi: 10.1111/j.1469-0691.2006.01479.x.

8. Gökengin D, Noori T, Alemany A, Bienkowski C, Liegon G, İnkaya AÇ, et al. Prevention strategies for sexually transmitted infections, HIV, and viral hepatitis in Europe. *Lancet Reg Health Eur*. 2023 Oct 26;34:100738. doi: 10.1016/j.lanepe.2023.100738.

9. Adamson PC, Loeffelholz MJ, Klausner JD. Point-of-care testing for sexually transmitted infections: A review of recent developments. *Arch Pathol Lab Med*. 2020 Nov 1;144(11): 1344-1351. doi: 10.5858/arpa.2020-0118-RA.

10. Elmi AA, Bansal D, Acharya A, Skariah S, Dargham SR, Abu-Raddad LJ, et al. Human papillomavirus (HPV) infection: molecular epidemiology, genotyping, seroprevalence and associated risk factors among Arab women in Qatar. *PLoS One*. 2017 Jan 3;12(1):e0169197. doi: 10.1371/journal.pone.0169197.

11. Finan RR, Irani-Hakime N, Tamim H, Almawi WY. Molecular diagnosis of human papillomavirus: comparison between cervical and vaginal sampling. *Infect Dis Obstet Gynecol*. 2001;9(2):119-22. doi: 10.1155/S1064744901000217.

12. World Health Organization. Global and regional sexually transmitted infection estimates for 2020. Available from https://www.who.int/data/gho/data/themes/topics/global-and-regional-sti-estimates. Accessed on December 18, 2023. World Health Organization 2023.

13. Kombe Kombe AJ, Li B, Zahid A, Mengist HM, Bounda GA, Zhou Y, et al. Epidemiology and burden of human papillomavirus and related diseases, molecular pathogenesis, and vaccine evaluation. *Front Public Health*. 2021 Jan 20;8:552028. doi: 10.3389/fpubh.2020.552028. .

14. Smolak A, Chemaitelly H, Hermez JG, Low N, Abu-Raddad LJ. Epidemiology of Chlamydia trachomatis in the Middle East and north Africa: a systematic review, meta-analysis, and meta-regression. *Lancet Glob Health*. 2019 Sep;7(9):e1197-e1225. doi: 10.1016/S2214-109X(19)30279-7. P

15. Mumtaz GR, Chemaitelly H, AlMukdad S, Osman A, Fahme S, Rizk NA, et al. Status of the HIV epidemic in key populations in the Middle East and North Africa: knowns and unknowns. *Lancet HIV.* 2022 Jul;9(7):e506-e516. doi: 10.1016/S2352-3018(22)00093-5.

16. Chemaitelly H, Majed A, Abu-Hijleh F, Blondeel K, Matsaseng TC, Kiarie J, Toskin I, Abu-Raddad LJ. Global epidemiology of Neisseria gonorrhoeae in infertile populations: systematic review, meta-analysis and metaregression. *Sex Transm Infect*. 2021 Mar;97(2):157-169. doi: 10.1136/sextrans-2020-054515.

17. Finan RR, Chemaitelly H, Racoubian E, Aimagambetova G, Almawi WY. Genetic diversity of human papillomavirus (HPV) as specified by the detection method, gender, and year of sampling: a retrospective cross-sectional study. *Arch Gynecol Obstet*. 2023 May;307(5): 1469-1479. doi: 10.1007/s00404-022-06907-4.

18. Al-Awadhi R, Chehadeh W, Kapila K. Prevalence of human papillomavirus among women with normal cervical cytology in Kuwait. *J Med Virol*. 2011 Mar;83(3):453-60. doi: 10.1002/jmv.21981.

19. Zheng LL, Chen SF, Yang F, Wang WH, Xu C, Zheng LY. High-risk HPV prevalence and genotype distribution among women in Liaocheng, Shandong Province, China from 2016 to 2022. *Front Public Health*. 2023 Mar 30;11:1145396. doi: 10.3389/fpubh.2023.1145396.

20. Finan RR, Tamim H, Almawi WY. Identification of Chlamydia trachomatis DNA in human papillomavirus (HPV) positive women with normal and abnormal cytology. *Arch Gynecol Obstet*. 2002 Jul;266(3):168-71. doi: 10.1007/s00404-001-0261-8.

21. López-Corbeto E, González V, Casabona J; Grupo de estudio CT NG. First data of Chlamydia trachomatis and other STI prevalence and co-infections in pregnant women under 25 years in Catalonia, Spain. *Med Clin (Barc).* 2021 Jan 8;156(1):33-34. doi: 10.1016/j.medcli.2019.12.019.

22. Kapiga S, Kelly C, Weiss S, Daley T, Peterson L, Leburg C, Ramjee G. Risk factors for incidence of sexually transmitted infections among women in South Africa, Tanzania, and Zambia: results from HPTN 055 study. *Sex Transm Dis*. 2009 Apr;36(4):199-206. doi: 10.1097/OLQ.0b013e318191ba01.

23 Skaletz-Rorowski A, Potthoff A, Nambiar S, Wach J, Kayser A, Kasper A, Brockmeyer NH. Age specific evaluation of sexual behavior, STI knowledge and infection among asymptomatic

adolescents and young adults. *J Infect Public Health*. 2020 Aug;13(8):1112-1117. doi: 10.1016/j.jiph.2020.04.005.

24. Adebamowo SN, Ma B, Zella D, Famooto A, Ravel J, Adebamowo C; ACCME Research Group. Mycoplasma hominis and Mycoplasma genitalium in the Vaginal Microbiota and Persistent High-Risk Human Papillomavirus Infection. *Front Public Health*. 2017 Jun 26;5:140. doi: 10.3389/fpubh.2017.00140.

25. Babi A, Issa T, Issanov A, Akhanova S, Udalova N, Koktova S, et al. Knowledge and attitudes of mothers toward HPV vaccination: A cross-sectional study in Kazakhstan. *Womens Health (Lond)*. 2023 Jan-Dec;19:17455057231172355. doi: 10.1177/17455057231172355.

Figure Legends

Figure 1. Prevalence of active infection with sexually transmitted infections among women gynecology clinic attendees in Beirut, Lebanon.

Figure 2. Prevalence of active infection with sexually transmitted infections A) by age group, B) smoking status, C) marital status, and D) number of sexual partners among women gynecology clinic attendees in Beirut, Lebanon.

Table 1

Characteristics of Study Participants

Characteristics	Categories	N (%)
Total sample size		351 (100.0)
Age (years)	Median (IQR) [1]	33.0 (29.0-37.0)
Age—years	20-29 years	100 (28.5)
20-29 years	30-39 years [2]	191 (54.4)
30-39 years*	40-49 years	49 (14.0)
40-49 years	50+ years	11 (3.1)
Smoking		110 (31.2)
Marital	Married	240 (68.4)
	Single/Divorced/Separated [3]	111 (31.6)
Sexually active		334 (95.2)
Numbers of partners	0-1 partner [4]	206 (58.7)
	2 partners	56 (15.9)
	3+ partners	89 (25.4)
HSV-1		0 (0.0)
HSV-2		2 (0.6)
Neisseria gonorrhoeae		1 (0.3)
Chlamydia trachomatis		10 (2.8)
Trichomonas vaginalis		0 (0.0)
Treponema pallidum		0 (0.0)
Mycoplasma hominis		72 (20.5)
Mycoplasma genitalium		7 (2.0)
HPV-High risk		55 (15.7)
HPV-Low risk		43 (12.2)
Candida albicans		48 (13.7)
Ureaplasma		24 (6.8)

IQR, interquartile range.

1. 8 observations were imputed at the median age of 33 years.
2. Includes 6 missing observations.
3. Includes 7 missing observations.
4. Includes 8 missing observations

Figure 1

Click here to access/download;Figure;HPV STD Fig. 1.docx

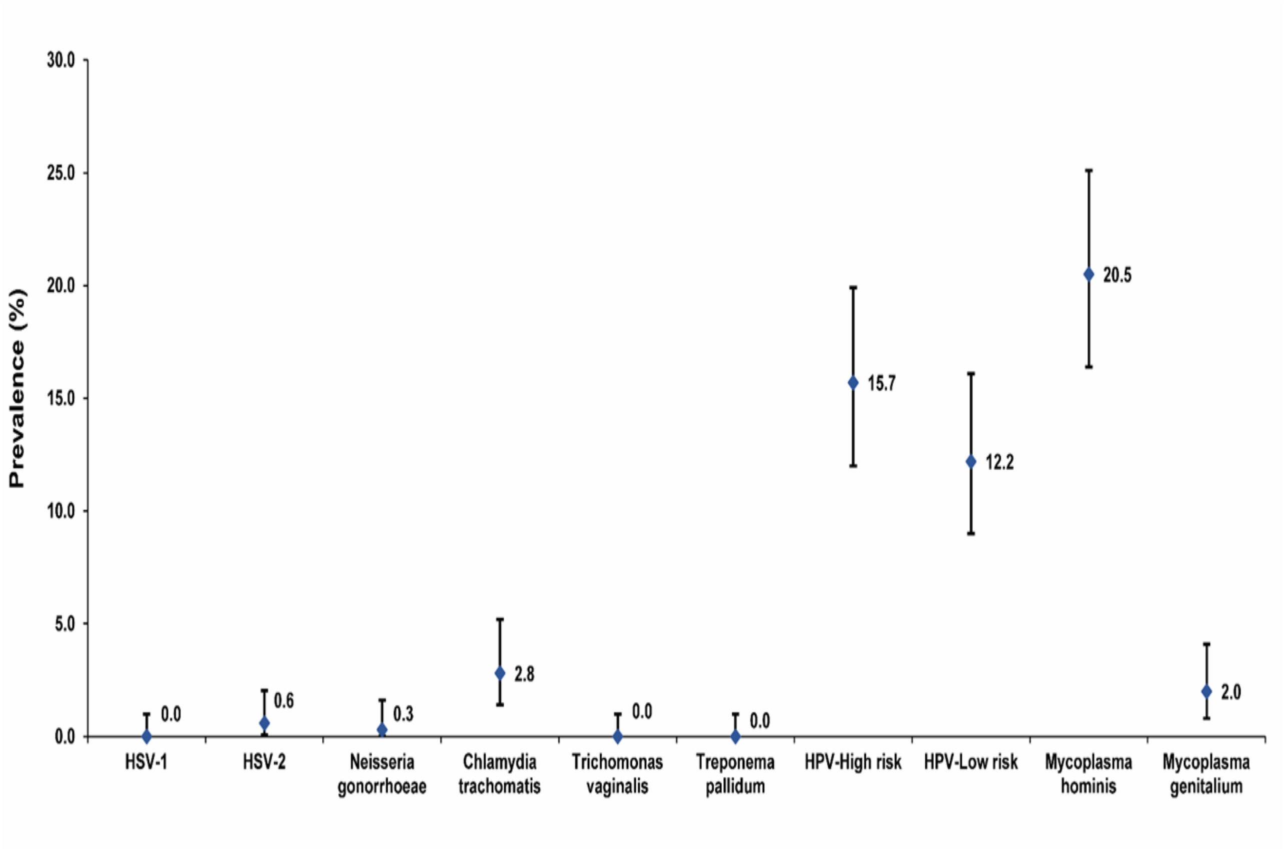

Figure 2

Click here to access/download;Figure;HPV STD Fig. 2.docx

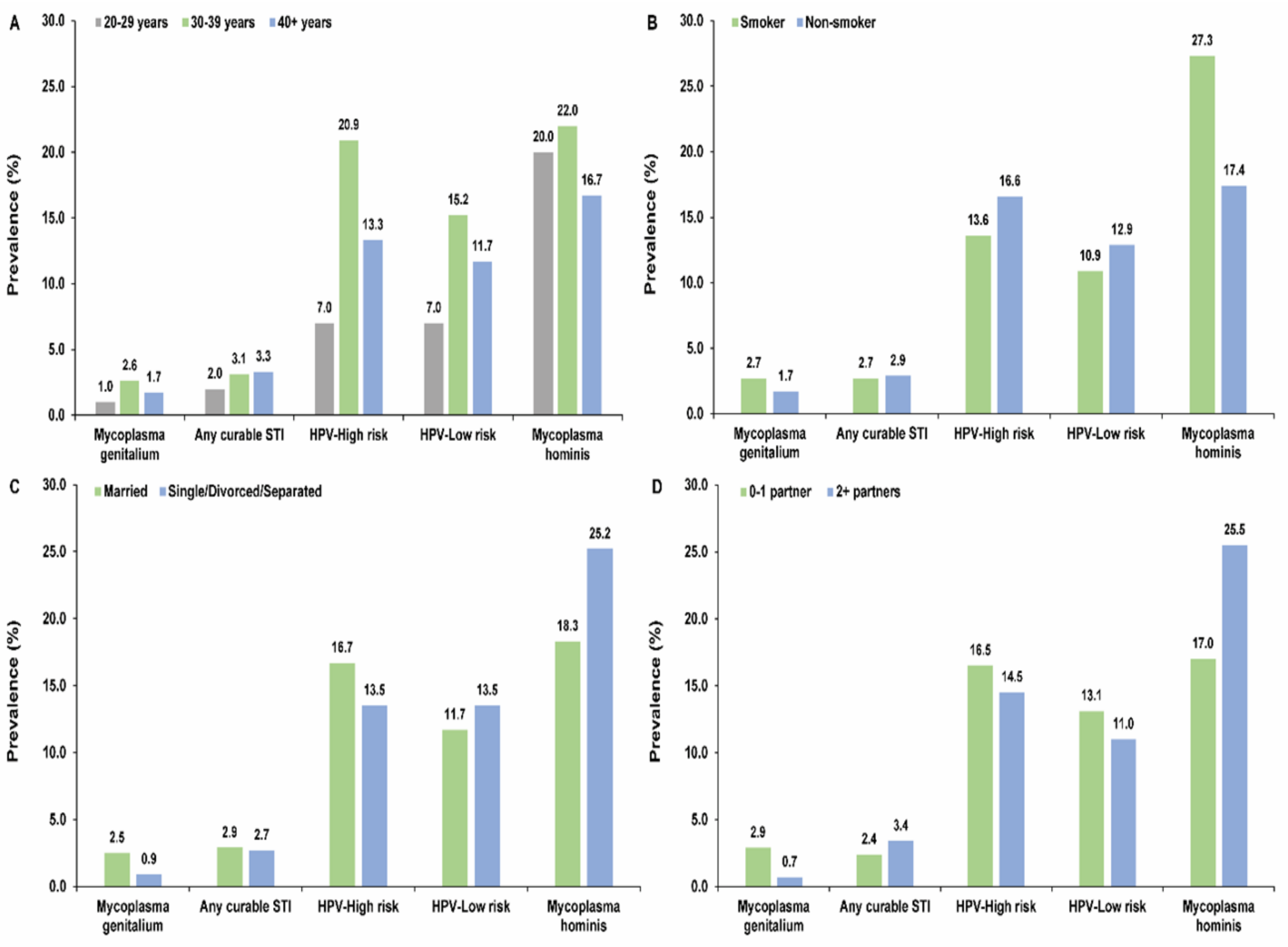

Any curable STI was defined as an active infection with any of *Neisseria gonorrhoeae*, *Chlamydia trachomatis*, *Trichomonas vaginalis*, and *Treponema pallidum*.

Supplementary Table 1

Click here to access/download
Supporting Information
HPV STD Table S1.docx

Supplementary Table 2

Click here to access/download
Supporting Information
HPV STD Table S2.docx

Supplementary Table 3

Click here to access/download
Supporting Information
HPV STD Table S3.docx

Supplementary Table 4

Click here to access/download
Supporting Information
HPV STD Table S4.docx

STROBE checklist

Click here to access/download
Supporting Information
PLOSOne_Human_Subjects_Research_Checklist.docx

Click here to access/download
Supporting Information
Ethics approval Almawi.pdf
